# Educational outcomes among children with congenital heart disease compared to peers: a Scotland-wide record-linkage study of 715,850 schoolchildren

**DOI:** 10.1186/s12887-024-04848-2

**Published:** 2024-06-22

**Authors:** Michael Fleming, Paul Athanasopoulos, Daniel F Mackay, Jill P Pell

**Affiliations:** School of Health and Wellbeing, University of Glasgow, Clarice Pears Building, 90 Byres Road, Glasgow, G12 8TB UK

**Keywords:** Congenital heart disease, Record linkage, Population study, Educational outcomes

## Abstract

**Background:**

Nine in every thousand children born in the United Kingdom have congenital heart disease, and 250,000 adults are living with the condition. This study aims to investigate the associations between congenital heart disease and educational outcomes among school-aged children in Scotland.

**Methods:**

Routine health and education databases were linked to produce a cohort of all singleton children born in Scotland and attending a local authority run primary, secondary, or special school in Scotland at some point between 2009 and 2013. Children with congenital heart disease within this cohort were compared with children unaffected by congenital conditions. Outcomes investigated were special educational need (SEN), absenteeism, exclusion, academic attainment, and unemployment. All analyses were adjusted for sociodemographic and maternity confounders. Absenteeism was investigated as a mediating factor in the associations with attainment and unemployment.

**Results:**

Of the 715,850 children, 6,295 (0.9%) had congenital heart disease and 4,412 (6.1%) had isolated congenital heart disease. Congenital heart disease and isolated congenital heart disease were both significantly associated with subsequent special educational need (OR 3.45, 95% CI 3.26–3.65, *p* < 0.001 and OR 1.98, 95% CI 1.84–2.13, *p* < 0.001 respectively), absenteeism (IRR 1.13, 95% CI 1.10–1.16, *p* < 0.001 and IRR 1.10, 95% CI 1.06–1.13, *p* < 0.001 respectively), and low academic attainment (OR 1.69, 95% CI 1.39–2.07, *p* < 0.001 and OR 1.35, 95% CI 1.07–1.69, *p* = 0.011 respectively). Neither congenital heart disease nor isolated congenital heart disease were associated with school exclusion. Only congenital heart disease (OR 1.21, 95% CI 1.03–1.42, *p* = 0.022) but not isolated congenital heart disease was associated with unemployment. When days absent were included in the analyses investigating attainment and unemployment, the conclusions were not altered.

**Conclusion:**

Children with congenital heart disease have greater special educational need, lower school attendance, attain lower examination grades and have greater unemployment compared to peers. In addition to healthcare support, affected children need educational support to avoid additional impact on their long-term wellbeing.

## Introduction

Congenital heart disease is the commonest form of birth defect with an incidence of nine cases per thousand live births in the United Kingdom [1] and United States of America [2]. It covers a wide spectrum of conditions including atrial and ventricular septal defects, tetralogy of Fallot, transposition of the great arteries, coarctation of the aorta, and hypo-plastic left heart syndrome [2]. In some, but not all, cases congenital heart disease occurs as part of a wider syndrome, such as Trisomy 21, Turner Syndrome, and DiGeorge Syndrome. The severity of congenital heart disease varies but up to one-quarter of cases have been classified as critical [3, 4], and 30% of infant deaths from birth defects are associated with a heart defect [2]. However, peripartum ultrasound [5], postnatal pulse-oximetry and advances in surgical treatment have improved early detection and survival [6–10] and 250,000 adults are currently living with congenital heart disease in the United Kingdom [11], as are two million Americans [3]. 

Congenital heart disease has been linked to poorer neurodevelopment and increased educational difficulty including special educational need (SEN) [12–15]. Other than one very small study [16], previous studies have suggested worse educational outcomes in all [17–22] or some [23, 24] of the children with congenital heart disease or for some, but not all, of the outcomes measured [25, 26]. However, studies on the educational outcomes of children with congenital heart disease have applied different outcome measures including battery tests, highest educational level, routinely administered standardised testing and examination grades. They have often been selective. Some have restricted inclusion to children with more severe conditions such as cyanotic heart disease [20, 27] or those requiring surgery in childhood [16, 17, 20, 21, 25, 27], and they are heterogeneous in terms of whether they exclude or include children with syndromes, chromosomal or genetic conditions, and comorbid non-cardiovascular congenital conditions.

This study uses population-wide linked national routine health and education data to compare school attendance (absenteeism and exclusion), special educational need, examination attainment and post-school unemployment among children with congenital heart disease with their peers.

## Methods

A retrospective cohort study was conducted on singleton children born in Scotland who attended a local authority run primary, secondary, or special school in Scotland at some point between 2009 and 2013 inclusive. The cohort was constructed by linking, at an individual level, health databases held by Public Health Scotland (PHS) and education databases held by the Scottish Exchange of Educational Data (ScotXed). The health databases covered maternity (Scottish Morbidity Record 02 (SMR02)) records, Scottish Birth Records (SBR), and admissions to neonatal units (SMR11) and general acute wards (SMR01). Within all SMR databases, diseases are recorded using the International Classification of Diseases versions 9 and 10 (ICD-9/ICD-10). The pupil census, conducted annually by all local authority run primary, secondary, and special schools across Scotland, records demographic data including whether a child has a special educational need including type. Absences and exclusions are appended at the end of the school year. The Scottish Qualifications Authority (SQA) collects examination attainment data for all Scottish schoolchildren. The school leaver database records pupils’ destination six months post-school: paid/voluntary employment, higher/further education, training, or unemployment.

The exposure of interest was congenital anomalies of the circulatory system (congenital heart disease), defined as any SMR01, SMR02, SBR or SMR11 record with a relevant recorded ICD code (ICD-9 745–747 or ICD-10 Q20-Q28). Children with any recorded congenital anomaly (ICD-9 740–759 or ICD-10 Q00-Q99) were also ascertained using the same datasets so that children without congenital heart disease who had another congenital anomaly could be excluded from the study. This ensured that the comparison group comprised of children with no congenital anomaly of any type. The outcomes of interest were special educational need, number of absences per year, number of exclusions per year, educational attainment, and unemployment. Annual school outcomes were number of days absent, number of exclusions for challenging/disruptive behaviour, and any record of SEN. Attendance data were only collected in years 2009, 2010, and 2012. Absenteeism across the school year was derived from half-days absent and expressed as full day equivalents. It included both authorised (ill health, work experience, authorised family vacations), and unauthorised (temporary exclusions, truancy, and unauthorised family vacations) absences. SEN is defined as unable to benefit from school education without help beyond that normally given to schoolchildren of similar age.

Final school leaver outcomes were academic achievement and unemployment six months post-school. Attainment was measured using the Scottish Credit Qualifications Framework which produces a composite measure of pupil performance across all national examinations, usually undertaken between 14 and 18 years of age. Attainment was treated as a binary variable: low attainment (≥ 1 at SCQF level 2, ≥ 5 at SCQF level 3, ≥ 2 but ≤ 7 at SCQF level 4 or > 0 but ≤ 4 at SCQF level 5) or high attainment (> 7 at SCQF level 4, ≥ 5 at SCQF level 5, ≥ 3 at SCQF level 6 or ≥ 1 at SCQF level 7). Destination six months post-school was collapsed into a dichotomous variable: education/employment/training versus unemployment.

Potential confounders included demographic (sex, age, ethnicity, socioeconomic deprivation), maternal (age at delivery, smoking status during pregnancy), and obstetric (parity, 5-minute Apgar score, mode of delivery, gestational age at delivery, and sex-gestation-specific birth weight centile) factors. Area-based socioeconomic deprivation was derived from the pupil’s postcode of residence, using the Scottish Index of Multiple Deprivation (SIMD) 2012, expressed as population quintiles.

Characteristics were summarised stratified by the presence or absence of congenital heart disease using frequencies and percentages for categorical data and means and standard deviations for continuous variables. The groups were compared using t-tests for continuous variables, χ^2^ tests for categorical variables and χ^2^ tests for trend for ordinal variables.

SEN, absences, and exclusions were analysed as yearly outcomes using population-averaged generalised estimating equations (GEE) adjusting for correlations between observations relating to the same pupil across different census years [28]. The user written quasi-likelihood under the independence model criterion (QIC) statistic compared different correlation structures. The structure with the lowest trace QIC was selected as most appropriate. An independent correlation structure, where the correlation between each measured variable is assumed to be zero, was found to be most appropriate in this analysis [28]. Number of days absent, and number of exclusions were modelled using longitudinal GEE analyses with a negative binomial distribution and log link function. Negative binomial regression was selected for this analysis because it allows for modelling of count variables when the data are over-dispersed [29]. Number of possible annual attendances was used as an offset variable when investigating both outcomes to adjust for individual exposure time. SEN was a dichotomous outcome and was modelled using GEE analyses with a binomial distribution and logit link function.

The associations between congenital heart disease and educational attainment and unemployment were investigated using binary logistic regression analyses univariately, then adjusted for sociodemographic and then maternal and obstetric confounders. For the sub-group of children with valid attendance data, the percentage of days absent across the full study period was calculated for each child and was then included as a covariate to test whether absenteeism mediated the respective associations.

The models were performed comparing all children with congenital heart disease against children with no congenital anomaly of any type. The models were then repeated after further excluding children with congenital heart disease who also had concurrent congenital abnormalities. This enabled the remaining children with isolated congenital heart disease to be compared against children with no congenital anomaly of any type. All models were run univariately, adjusted for sociodemographic factors, and then also adjusted for maternal and obstetric factors. The analyses were undertaken using R Core Team (2017) and Stata MP version 14. The study was approved by the National Health Service (NHS) Public Benefit and Privacy Panel (reference 1920 − 0144).

## Results

The full cohort comprised of 715,850 pupils, 6,295 (0.88%) of whom had congenital heart disease, and 4,412 (0.62%) had isolated congenital heart disease. Compared with their peers, children with congenital heart disease were more likely to be deprived, born preterm, born to mothers who smoked during pregnancy, delivered by Caesarean section, have lower 5-minute Apgar scores, and have lower sex- gestation-specific birth weight centiles irrespective of whether they also had other congenital anomalies (Table 1). Each child in the cohort had between one and five years of data available.

**Table 1:** Characteristics of singleton schoolchildren with and without congenital heart disease

		No congenital anomalies		Congenital heart disease			Isolated congenital heart disease		
		*N* = 709,555		*N* = 6,295			*N* = 4,412		
		*n*	%	*n*	%		*n*	%	
**Age**						< 0.001			< 0.001
	mean (SD)	10.91 (3.97)		10.55 (3.81)			10.55 (3.78)		
**Sex**						0.012			0.237
	male	355,003	*50.0*	3,250	*51.6*		2,168	*49.1*	
	female	354,552	*50.0*	3,045	*48.4*		2,244	*50.9*	
	Missing	0		0			0		
**Deprivation Quintile**						< 0.001			0.010
	1 = most deprived	161,180	*22.7*	1,537	*24.4*		1,069	*24.2*	
	2	142,172	*20.1*	1,284	*20.4*		891	*20.2*	
	3	137,135	*19.3*	1,200	*19.1*		835	*18.9*	
	4	138,727	*19.6*	1,216	*19.3*		856	*19.4*	
	5 = least deprived	129,793	*18.3*	1,051	*16.7*		758	*17.2*	
	Missing	548		7			3		
**Ethnic group**						0.489			0.650
	White	672,235	*96.3*	5,984	*96.3*		4,205	*96.7*	
	Asian	16,089	*2.3*	147	*2.4*		90	*2.1*	
	Black	1,752	*0.3*	17	*0.3*		10	*0.2*	
	Mixed	6,079	*0.9*	49	*0.8*		35	*0.8*	
	Other	1,844	*0.3*	14	*0.2*		8	*0.2*	
	Missing	11,556		84			64		
**Maternal age (years)**						0.045			0.092
	<=24	194,365	*27.4*	1,782	*28.3*		1,299	*29.4*	
	24–29	208,129	*29.3*	1,752	*27.8*		1,273	*28.9*	
	30–34	201,021	*28.3*	1,633	*25.9*		1,135	*25.7*	
	>=35	106,029	*14.9*	1,127	*17.9*		704	*16.0*	
	Missing	11		1			1		
**Maternal smoking**						< 0.001			0.002
	No	454,843	*64.1*	3,875	*61.6*		2,766	*62.7*	
	Yes	173,168	*24.4*	1,704	*27.1*		1,173	*26.6*	
	Unknown	81,544	*11.5*	716	*11.4*		473	*10.7*	
	Missing	0		0			0		
**Mode of delivery**						< 0.001			< 0.001
	spontaneous vaginal delivery	466,153	*65.7*	3,409	*54.2*		2,448	*55.5*	
	assisted vaginal	15,371	*2.2*	90	*1.4*		71	*1.6*	
	breech vaginal	84,579	*11.9*	634	*10.1*		463	*10.5*	
	elective caesarean section	1,889	*0.3*	101	*1.6*		66	*1.5*	
	emergency caesarean section	53,252	*7.5*	646	*10.3*		447	*10.1*	
	other	88,156	*12.4*	1,415	*22.5*		917	*20.8*	
	Missing	155		0			0		
**Gestation (weeks)**						< 0.001			< 0.001
	<=24	77	*0.0*	46	*0.7*		29	*0.7*	
	25	81	*0.0*	62	*1.0*		38	*0.9*	
	26	181	*0.0*	123	*2.0*		81	*1.8*	
	27	275	*0.0*	118	*1.9*		82	*1.9*	
	28	508	*0.1*	150	*2.4*		109	*2.5*	
	29	646	*0.1*	111	*1.8*		88	*2.0*	
	30	1,042	*0.1*	93	*1.5*		71	*1.6*	
	31	1,291	*0.2*	93	*1.5*		62	*1.4*	
	32	2,072	*0.3*	93	*1.5*		62	*1.4*	
	33	2,886	*0.4*	89	*1.4*		59	*1.3*	
	34	5,075	*0.7*	136	*2.2*		73	*1.7*	
	35	8,266	*1.2*	160	*2.5*		84	*1.9*	
	36	15,365	*2.2*	269	*4.3*		157	*3.6*	
	37	34,229	*4.8*	427	*6.8*		250	*5.7*	
	38	88,375	*12.5*	816	*13.0*		527	*12.0*	
	39	147,405	*20.8*	1,107	*17.6*		789	*17.9*	
	40	215,495	*30.4*	1,270	*20.2*		985	*22.3*	
	41	159,689	*22.5*	969	*15.4*		737	*16.7*	
	>=42	26,073	*3.7*	160	*2.5*		126	*2.9*	
	Missing	524		3			3		
**5-min Apgar score**						< 0.001			< 0.001
	0–3	3,230	*0.5*	111	*1.8*		59	*1.3*	
	4–6	6,380	*0.9*	246	*3.9*		141	*3.2*	
	7–10	692,818	*97.6*	5,788	*91.9*		4,106	*93.1*	
	Missing	7,127		150			106		
**Sex-gestation-specific birthweight centile**						< 0.001			0.003
	1–3	28,416	*4.0*	500	*7.9*		253	*5.7*	
	4–10	63,120	*8.9*	711	*11.3*		429	*9.7*	
	11–20	84,568	*11.9*	781	*12.4*		550	*12.5*	
	21–80	418,247	*58.9*	3,256	*51.7*		2,400	*54.4*	
	81–90	60,587	*8.5*	517	*8.2*		396	*9.0*	
	91–97	38,093	*5.4*	338	*5.4*		257	*5.8*	
	98–100	15,652	*2.2*	167	*2.7*		112	*2.5*	
	Missing	872		25			15		
**Parity**						0.167			0.785
	0	318,675	*44.9*	2,812	*44.7*		2,001	*45.4*	
	1	245,620	*34.6*	2,106	*33.5*		1,494	*33.9*	
	2 or more	141,812	*20.0*	1,331	*21.1*		889	*20.1*	
	Missing	3,448		46			28		

Among the 6,295 children with congenital heart disease and 4,412 children with isolated congenital heart disease, 2,382 (37.8%) and 1,198 (27.2%) respectively had a special educational need compared with 101,918 (14.4%) among 709,555 children without congenital anomalies. Congenital heart disease and isolated congenital heart disease were both significantly associated with subsequent special educational need after adjusting for demographic, maternal and obstetric factors (OR 3.45, 95% CI 3.26–3.65, *p* < 0.001 and OR 1.98, 95% CI 1.84–2.13, *p* < 0.001 respectively) (Table 2).

**Table 2:** Generalized estimating equation models of the associations between congenital heart disease and (**a**) school absence, (**b**) school exclusion, and (**c**) special educational need

		Univariate		Adjusted for demographic factors		Adjusted for demographic, maternal, and obstetric factors	
	Number of records (pupils)	IRR (95% CI)	*P* value	IRR (95% CI)	*P* value	IRR (95% CI)	*P* value
School Absence							
No congenital anomaly	1,453,452 (638,872)	1.00		1.00		1.00	
Congenital heart disease	12,725 (5,574)	1.12 (1.08–1.15)	< 0.001	1.15 (1.12–1.18)	< 0.001	1.13 (1.10–1.16)	< 0.001
Isolated congenital heart disease	8,922 (3,909)	1.08 (1.04–1.12)	< 0.001	1.12 (1.08–1.15)	< 0.001	1.10 (1.06–1.13)	< 0.001
School Exclusion							
No congenital anomaly	1,453,452 (638,872)	1.00		1.00		1.00	
Congenital heart disease	12,725 (5,574)	1.00 (0.84–1.20)	0.963	0.97 (0.82–1.15)	0.745	0.95 (0.80–1.13)	0.56
Isolated congenital heart disease	8,922 (3,909)	1.04 (0.84–1.28)	0.727	1.04 (0.85–1.27)	0.676	1.04 (0.85–1.27)	0.711
	**Number of records (pupils)**	**OR (95% CI)**	***P*** ** value**	**OR (95% CI)**	***P*** ** value**	**OR (95% CI)**	***P*** ** value**
Special Educational Need							
No congenital anomaly	2,534,902 (697,697)	1.00		1.00		1.00	
Congenital heart disease	22,914 (6,072)	3.89 (3.69–4.11)	< 0.001	3.98 (3.77–4.21)	< 0.001	3.45 (3.26–3.65)	< 0.001
Isolated congenital heart disease	16,125 (4,262)	2.22 (2.07–2.38)	< 0.001	2.27 (2.11–2.44)	< 0.001	1.98 (1.84–2.13)	< 0.001

Demographic factors: age, sex, deprivation quintile and ethnic group

Maternal and obstetric factors: maternal smoking during pregnancy, maternal age, parity, mode of delivery, 5-minute Apgar score, gestational age, and sex- gestation-specific birth weight centile

IRR incidence rate ratio; OR odds ratio; CI confidence interval

School attendance data were available for 655,930 pupils, of whom 5,776 (0.88%) had congenital heart disease, and 4,049 (0.62%) had isolated congenital heart disease. Average number of days absent per year were higher among the 5,776 children with congenital heart disease (mean 13.2 days; median 9 days) and 4,049 children with isolated congenital heart disease (mean 12.7 days; median 8.5 days) compared to 650,154 children without congenital anomalies (mean 11.7 days; median 7.5 days). Congenital heart disease and isolated congenital heart disease were both significantly associated with school absence after adjusting for demographic, maternal and obstetric factors (IRR 1.13, 95% CI 1.10–1.16, *p* < 0.001 and IRR 1.10, 95% CI 1.06–1.13, *p* < 0.001 respectively) (Table 2). Among the 5,776 children with congenital heart disease and 4,049 children with isolated congenital heart disease, 218 (3.8%) and 157 (3.9%) respectively were excluded from school at least once over the study period compared with 24,660 (3.8%) among 650,154 children without congenital anomalies. Neither congenital heart disease or isolated congenital heart disease were significantly associated with school exclusion (Table 2).

Educational attainment data were available for 131,181 pupils, 822 (0.63%) of whom had congenital heart disease, and 639 (0.49%) had isolated congenital heart disease.

Among the 822 children with congenital heart disease and 639 children with isolated congenital heart disease, 358 (43.6%) and 263 (41.2%) respectively achieved low level of attainment compared with 46,765 (35.9%) among 130,359 children without congenital anomalies. The association between congenital heart disease and low academic attainment was significant on univariate analysis and remained so after adjusting for demographic, maternal and obstetric confounders (OR 1.69, 95% CI 1.39–2.07, *p* < 0.001) (Table 3). Isolated congenital heart disease was significantly associated with lower educational attainment on univariate analysis and remained so after adjusting for demographic, maternal and obstetric factors (OR 1.35, 95% CI 1.07–1.69, *p* = 0.011) (Table 3). When the logistic regression models for educational attainment were re-run using only the sub-group of children with school absence data, inclusion of school absence as a covariate attenuated the effect estimates however the association between congenital heart disease and educational attainment remained statistically significant (Table 3).

**Table 3:** Binary logistic regression models of the associations between congenital heart disease and (**a**) low educational attainment and (**b**) unemployment

		Univariate		Adjusted for demographic factors		Adjusted for demographic, maternal, and obstetric factors		Adjusted for demographic, maternal, and obstetric factors and days absent	
	Number of pupils	OR (95% CI)	*P* value	OR (95% CI)	*P* value	OR (95% CI)	*P* value	OR (95% CI)	*P* value
Low educational attainment									
No congenital anomaly	130,023	1.00		1.00		1.00		-	
Congenital heart disease	821	1.38 (1.20–1.58)	< 0.001	1.77 (1.46–2.15)	< 0.001	1.69 (1.39–2.07)	< 0.001	-	-
Isolated congenital heart disease	638	1.25 (1.06–1.46)	0.006	1.40 (1.12–1.76)	0.003	1.35 (1.07–1.69)	0.011	-	-
Low educational attainment among subgroup of children with valid absence data									
No congenital anomaly	126,342	1.00		1.00		1.00		1.00	
Congenital heart disease	792	1.38 (1.19–1.58)	< 0.001	1.79 (1.47–2.18)	< 0.001	1.72 (1.40–2.10)	< 0.001	1.59 (1.29–1.96)	< 0.001
Isolated congenital heart disease	613	1.23 (1.04–1.44)	0.013	1.40 (1.12–1.76)	0.004	1.35 (1.07–1.71)	0.011	1.27 (1.00-1.62)	0.05
Unemployment									
No congenital anomaly	203,148	1.00		1.00		1.00		-	
Congenital heart disease	1,404	1.31 (1.12–1.53)	0.001	1.24 (1.06–1.46)	0.008	1.21 (1.03–1.42)	0.022	-	-
Isolated congenital heart disease	1,008	1.10 (0.90–1.33)	0.362	1.02 (0.83–1.24)	0.879	1.01 (0.82–1.23)	0.944	-	-
Unemployment among subgroup of children with valid absence data									
No congenital anomaly	193,942	1.00		1.00		1.00		1.00	
Congenital heart disease	1,333	1.32 (1.13–1.55)	0.001	1.26 (1.06–1.48)	0.007	1.22 (1.03–1.44)	0.02	1.22 (1.03–1.44)	0.024
Isolated congenital heart disease	955	1.10 (0.90–1.35)	0.348	1.02 (0.83–1.26)	0.837	1.01 (0.82–1.25)	0.925	1.00 (0.81–1.24)	0.983

Demographic factors: age, sex, deprivation quintile and ethnic group

Maternal and obstetric factors: maternal smoking during pregnancy, maternal age, parity, mode of delivery, 5-minute Apgar score, gestational age, and sex- gestation-specific birth weight centile

OR odds ratio; CI confidence interval

Leaver destination data were available for 205,252 pupils, 1,413 (0.69%) of whom had congenital heart disease, and 1,016 (0.50%) had isolated congenital heart disease. Among the 1,413 children with congenital heart disease and 1,016 children with isolated congenital heart disease, 186 (13.2%) and 115 (11.3%) respectively were unemployed 6 months after leaving school compared with 21,081 (10.3%) among 203,839 children without congenital anomalies. The association between congenital heart disease and unemployment was significant on univariate analysis and remained so after adjusting for demographic, maternal and obstetric confounders (OR 1.21, 95% CI 1.03–1.42, *p* =0.002) (Table 3). There was no association between isolated congenital heart disease and unemployment on univariate analysis or after adjusting for demographic, maternal and obstetric factors (OR 1.01, 95% CI 0.82–1.23, *p* = 0.944) (Table 3). When the logistic regression models for unemployment were re-run using only the sub-group of children with school absence data, inclusion of school absence as a covariate did not alter the results as the associations with congenital heart disease and isolated congenital heart disease remained significant (OR 1.22, 95% CI 1.03–1.44, *p* = 0.024) and non-significant (OR 1.00, 95% CI 0.81–1.24, *p* = 0.983) (respectively. (Table 3).

## Discussion

In a national cohort of singleton schoolchildren, congenital heart disease was shown to be associated with higher absenteeism from school, increased special educational need, and lower educational attainment, independent of demographic, maternal and obstetric factors, and irrespective of comorbid congenital anomalies. Congenital heart disease, but not isolated congenital heart disease, was associated with increased post-school unemployment. Higher absenteeism may play a role in partially mediating the association with lower educational attainment.

Our findings regarding academic attainment are consistent with previous studies that have generally reported poorer educational outcomes among children with congenital heart disease. Studies using battery tests have been conducted on relatively small sample sizes; usually fewer than 50 children [16, 19, 20, 25, 26] and have focused on more severe cases of congenital heart disease, such as cyanotic heart disease and disease requiring surgery in childhood. A retrospective cohort study, conducted in the USA, reported lower battery test scores among 28 children who had previous surgery for hypoplastic left heart syndrome than age-matched controls [19]. Another USA study reported no significant difference overall between 91 children with tetralogy of Fallot and 87 children without congenital heart disease, but significantly lower reading and mathematics scores in the sub-group with syndromes [23]. An Australian study compared 29 children with surgically corrected tetralogy of Fallot or transposition of the great arteries with children with cardiac murmurs not requiring treatment and reported significantly worse scores in the domains of reading, spelling, and arithmetic [20]. 

A small USA study compared 13 children who had had surgery for ventricular septal defects, an acyanotic condition, with matched controls [16]. Children with syndromes and multiple genetic abnormalities were excluded. They reported that the scores for reading, spelling, mathematics, and IQ were comparable to population norms. In an Australian prospective cohort study of 21 children who had surgical repair of cyanotic or acyanotic heart disease, excluding syndromes, battery tests demonstrated worse numeracy but no difference in verbal comprehension, spelling, reading, or mathematics [25]. In a British prospective cohort study, compared with children requiring bone marrow transplants, 47 children with congenital heart disease performed worse prior to surgery with no difference after surgery. Prior to surgery children with cyanotic heart disease performed worse than those with acyanotic heart disease in reading, spelling, and arithmetic on the British Assessment Scale (BAS). Post-surgery they still performed worse for the first two [26]. 

A Swiss study used questionnaires to ascertain educational attainment in a hospitalised cohort [24]. Overall, educational attainment among 207 adolescents with congenital heart disease did not differ significantly from the general population. However, attainment was significantly lower among the sub-group with severe disease [24]. 

Studies using routine data on state-administered tests have been larger in scale. A retrospective population cohort study, conducted in New South Wales Australia, compared 396 children who had a cardiac procedure in the first year of life with children who had undergone other surgical procedures, excluding children with multiple congenital abnormalities or cerebral palsy [21]. In the state assessments, the former were more likely to be below national minimum standards for numeracy, grammar, punctuation, writing and spelling and less likely to be in the top three bands.

A retrospective cohort study recruited 362 children attending public schools in Arkansas USA who had surgery in infancy for congenital heart disease, but excluded children with genetic conditions, who had extreme low birth weight and the 5% of children who did not undertake state tests [17]. Compared with state norms, they had more than double the prevalence of special educational needs and lower rates of test proficiency in literacy and mathematics.

A retrospective population cohort study of 2,807 children with congenital heart disease in North Carolina, USA excluded children with chromosomal anomalies but included children with multiple congenital abnormalities [18]. Failure to meet test standards was more common among children with congenital heart disease than children with no structural birth defects, and more common among children with critical disease than those with non-critical disease.

In a Danish population cohort study, of 2,986 with congenital heart disease, completion of basic and secondary schooling and overall level of attainment were lower than in a comparison group, irrespective of whether extra-cardiac defects and chromosomal abnormalities were included and irrespective of whether the disease was severe or not [22]. There were no differences in vocational education.

Absenteeism has been reported to be higher among children with chronic illnesses [30] including congenital heart disease specifically [31], and higher absenteeism from school is associated with poorer performance on standardised testing [32, 33]. Therefore, it is plausible that poorer attainment, and resulting unemployment, among children with congenital heart disease may be a result of their higher absence rates. However, our findings suggest that absenteeism only partially explains lower educational attainment and unemployment at best.

Congenital heart defects are known to increase the risk of neurodevelopmental outcomes in children and adolescents [12, 13] however studies investigating specific outcomes have been rare. Pei-Chen Tsao et al. [34] found in a study in Taiwan that congenital heart defects were associated with a 2.5 fold increase in ADHD (95% CI 1.96–3.25) and a 1.97 fold increase in ASD (95% CI 1.11–3.52) while Riehle-Colarusso’s [14] study of a cohort of 860,000 children in Atlanta, USA found that children with congenital heart defects were 1.5 times more likely to receive special educational services than children without defects. Our effect size was of a similar magnitude when looking at isolated congenital heart disease.

Our study was conducted on a large, national cohort of children to maximise statistical power and representativeness. Use of routine data ensured complete coverage of all local authority-maintained schools: mainstream and special schools, and primary and secondary schools. Private schools could not be included but account for less than 5% of children in Scotland [35]. We were able to adjust for a wide range of potential confounding factors, including demographic, maternal and obstetric factors and were able to determine whether the association between congenital heart disease and lower educational attainment and unemployment could be explained by higher absenteeism. However, as with all observational studies, residual confounding is possible. The reasons for school absence were not available. We had sufficient statistical power to conduct sub-group analyses on children with isolated congenital heart disease in order ascertain if the associations were specifically attributed to this condition or comorbid congenital anomalies. However, the study was not powered to stratify children by type of congenital heart disease, category (e.g. cyanotic versus acyanotic) or severity. The outcomes – educational attainment, special educational need, leaver destination, exclusion, and absenteeism – were objectively measured, obviating reporting or recall bias. The study was restricted to singleton children because linkage of maternity records to offspring records is unreliable for same sex multiple births. The linkage of birth to educational records meant that pupils attending Scottish schools who were born outside of Scotland, and children who were born in Scotland but emigrated prior to commencing school, were not included. Finally, this was a single country analysis and therefore findings may not be generalizable to other countries.

In conclusion, children with congenital heart disease have poorer educational attainment, increased special educational need, and greater absenteeism irrespective of whether they also have other congenital anomalies. The increase in educational difficulty is not explained by confounding and, at best, is only partially mediated by their higher rates of school absence. These children are already disadvantaged by their health conditions and educational support is required to ensure that their poorer educational attainment does not disadvantage them further.

## Data Availability

The datasets generated and analysed during the study are not publicly available. All health data are owned by Public Health Scotland (https://www.publichealthscotland.scot), and all education data are owned by the Scottish Government (www2.gov.scot/Topics/Statistics/ScotXed). Under the terms of our data access agreements with them we are not permitted to pass the data onto third parties. Interested researchers may apply at these sites for data access to health and education data by emailing phs.edris@phs.scot and ASU_schools_Data_Access@gov.scot respectively. The authors applied for permission to access, link, and analyse these data and undertook mandatory training in data protection, IT security and information governance. The study was approved by the NHS Public Benefit and Privacy Panel and covered by a data processing agreement between Glasgow University and Public Health Scotland and a data sharing agreement between Glasgow University and ScotXed. The electronic Data Research and Innovation Service (eDRIS) within Public Health Scotland helped the authors obtain approvals, linked the data, and uploaded the final datasets into a secure analytical platform within the National Safe Haven for the researchers to analyse. The researchers did not receive any special privileges or access to the third-party data.
